# Signing with the Face: Emotional Expression in Narrative Production in Deaf Children with Autism Spectrum Disorder

**DOI:** 10.1007/s10803-018-3756-x

**Published:** 2018-09-28

**Authors:** Tanya Denmark, Joanna Atkinson, Ruth Campbell, John Swettenham

**Affiliations:** 1 Division of Psychology and Language Science, Department of Language and Cognition, University College London, London, UK; 2 Division of Psychology and Language Science, Deafness, Cognition and Language Research Centre, University College London, 2 Wakefield Street, Chandler House, London, WC1N 9PF UK

**Keywords:** Deaf, Autism, British Sign Language, Emotion, Narrative

## Abstract

This study examined facial expressions produced during a British Sign Language (BSL) narrative task (Herman et al., International Journal of Language and Communication Disorders 49(3):343–353, 2014) by typically developing deaf children and deaf children with autism spectrum disorder. The children produced BSL versions of a video story in which two children are seen to enact a language-free scenario where one tricks the other. This task encourages elicitation of facial acts signalling intention and emotion, since the protagonists showed a range of such expressions during the events portrayed. Results showed that typically developing deaf children produced facial expressions which closely aligned with native adult signers’ BSL narrative versions of the task. Children with ASD produced fewer targeted expressions and showed qualitative differences in the facial actions that they produced.

## Introduction

Deaf people use facial expressions while they are using sign language to express their own emotions or to describe the emotions of others, through the use of the same range of emotional facial expressions used naturally by the general population e.g. happiness, anger, sadness etc. (Carminati and Knoeferle 2013). They also use facial actions which provide sign language prosody, which function in sign language like intonation in spoken languages. For hearing speakers, prosody is carried in the vocal channel through patterns of stress, rhythm and intonation, while for deaf sign language users, prosody is conveyed while signing through an extensive range of prosodic facial acts conducted in synchrony with movements and holds produced by the hands (Dachovsky and Sandler 2009). Sign language prosody functions include lengthening effects, as well as lower face behaviours, eyeblinks and torso leans (Brentari and Crossley 2002). In addition, certain facial actions are also considered to be integral to providing phonological, lexical, syntactic and discourse features in sign language (e.g. Sutton-Spence and Woll 1999; Stokoe 2005; Elliott and Jacobs 2013). Neuropsychological studies of deaf signers with discrete right and left hemisphere lesions (Corina et al. 1999; MacSweeney et al. 2008) have demonstrated a dissociation between linguistic and non-linguistic uses of the face with linguistic functions being localized to the left hemisphere and affective functions being mediated by the right hemisphere.

Deaf signers have been shown to attend to faces to a greater extent than hearing people (Agrafiotis et al. 2006; Emmorey et al. 2009; Mitchell et al. 2013; Megreya and Bindemann 2017) and show face processing advantages relative to hearing non-signers (Bellugi et al. 1990; Bettger et al. 1997). It is plausible that one of the sources of this advantage relates to deafness itself, reflecting greater dependence on the visual channel for communication. Thus, deaf people attend more closely to facial actions which serve communication, whether or not they use sign language (see, for instance, Hauthal et al. 2012). However, since facial actions have developed within sign languages to a very marked extent, subserving linguistic as well as communicative functions (Campbell et al. 1999; Thompson et al. 2009), sign language exposure may play a further role in deaf people’s face processing abilities.

Research with hearing children diagnosed with autism spectrum disorder (ASD) has investigated both comprehension and production of emotional facial expressions. The majority of these studies have focused on emotion recognition, and while not all demonstrate impairment in emotion processing (Harms et al. 2010), there are many studies that do (e.g. Ashwin et al. 2006; Lindner and Rosen 2006; Dziobek et al. 2010; Greimel et al. 2010; Lartseva et al. 2014; Chen et al. 2015; Brewer et al. 2016; Hubbard et al. 2017). By contrast, there have been relatively few studies on the production of emotional expressions in children with ASD. Observational studies in naturalistic settings have shown atypical use of facial expressions (Yirmiya et al. 1989; Capps et al. 1993; Dawson et al. 1990; Kasari et al. 1990; Bieberich and Morgan 2004; Stagg et al. 2014). For example, Yirmiya et al., (1989) compared children with ASD and a matched control group with developmental delay (DD) in their use of emotional facial expressions using the Early Social Communication Scales (Mundy et al. 2003), a videotaped structured observational measure. Children with ASD displayed significantly less affect in their facial expressions compared to DD controls. Similarly, Capps et al., (1993) found that parents of children with ASD report that their children show less positive affect in their facial expressions compared to parents of typically developing (TD) children.

The production of facial expressions has also been examined in more controlled experimental studies. For example, MacDonald et al. (1989) took photographs of high functioning adults with ASD and neurotypical (NT) controls producing facial expressions for five different emotions (‘happy’, ‘sad’, ‘fear’, ‘angry’ and ‘neutral’). Judges (who were blind to experimental group) were asked to identify the emotion in these photos using a forced-choice rating system. The judges were significantly less likely to correctly identify the emotion expressed by individuals with ASD. Their facial expressions were rated as more “odd” relative to those produced by NT children. Volker et al. (2009) compared a group of 6–13 year old high-functioning children with ASD with matched TD children on their ability to enact facial expressions for six basic emotions (‘happy’, ‘sad’, ‘fear’, ‘anger’, ‘surprise’ and ‘disgust’). Participants in both groups were read a statement and asked to show a targeted emotion from the statement (e.g. “I want you to think of a time that you were really, really happy… show me a happy face.”). Six raters blind to group membership scored the children’s enacted facial expressions in terms of accuracy and oddity. Raters were less able to recognize expressions of sadness produced by children with ASD than by TD children, and facial expressions of the children with ASD were more likely to be rated as “odd” compared to TD expressions. More recently, Brewer et al., (2016) investigated ASD and NT adults’ ability to recognise emotional expressions produced by ASD and NT models. This was the first study to use raters with ASD in addition to NT raters. They found that ASD expressions were more poorly recognized than NT expressions regardless of recognizer group (ASD or NT), suggesting that the atypical emotional expressions are idiosyncratic, rather than systematic, and were not shared with other individuals with ASD. Together, these studies provide strong evidence that the production of facial expressions for emotion is impaired in ASD in the hearing population.

In addition to facial expression, several studies have demonstrated unusual prosody in speech production in ASD such as: monotone intonation (speech with narrow pitch range); rate of speech being too fast; limited or unusual pitch ranges; poor volume modulation; and more frequent errors in residual articulation distortion (Baltaxe and Guthrie 1987; Shriberg et al. 2001; Peppé et al. 2006; Hubbard and Trauner 2007). Grossman et al. (2013) compared young people with ASD and matched TD controls in their production of both vocal and facial expressions during a spoken narrative in a story retelling task. Raters blind to group membership coded the narrative videoclips for expressed emotion, intensity of expression, and naturalness/awkwardness of expression. Both groups produced vocal and facial expressions that were categorically accurate, but their productions were rated as being qualitatively different, with the ASD group producing fewer natural and more awkward vocal and facial expressions.

There have been very few studies investigating ASD in deafness (Quinto-Pozo et al. 2011; Hansen and Scott 2018). Studies of communication skills in deaf children with ASD have identified characteristics of their communication equivalent to those found in hearing children with ASD (Scawin 2003; Shield and Meier 2012; Szymanski et al. 2012; Shield et al. 2015, 2016, 2017). For example, confusing self and other in both gesture and use of pronouns occurs in hearing children with ASD (Lee et al. 1994). Similar patterns are observed in deaf signing children with ASD, who showed a tendency to reverse palm orientation on signs that must be specified for inward/outward orientation, as well as difficulty using American Sign Language pronouns, which tended to be avoided in favour of names (Shield and Meier 2012; Shield et al. 2015). In a study of comprehension of emotional facial expressions in sign language in deaf ASD and TD groups, we have found that the ASD group showed a deficit during sign language processing analogous to the deficit in vocal emotion recognition that has been observed in hearing children with ASD (Denmark et al. 2014).

Deaf individuals must attend to the face in order to communicate, but little is known about attention to faces in deaf people with ASD and whether or not they are impaired in aspects of sign language communication involving the face. Two case studies of deaf individuals with ASD have described impairments in the use of facial expressions. Poizner et al. (1990) investigated the signing skills of a deaf adult signer with ASD (Judith), who had been exposed to sign language from birth through communication with her deaf parents. Despite an optimal native signing environment she had production deficits, with a distinct absence of facial expressions or gesture in her signed output. Morgan et al. (2002) described a hearing linguistic savant who was described as having mild autism (Christopher) who showed fluency in multiple spoken languages. A qualified deaf tutor taught British Sign Language to Christopher and a comparison group of hearing BSL learners, with emphasis on the core grammatical properties of the language. At each stage, their progress was recorded. Christopher used minimal facial expressions when signing, and found the use of facial action difficult, especially its coordination with manual signing. This affected his ability to use facial actions to produce prosodically correct BSL. For instance, he was unable to indicate negation and question-type using his face. However, it should be noted that these are single case observations, so we should be cautious in generalising the findings to a larger population.

Most studies examining the production of facial expressions in hearing people use controlled methods to elicit expressions; participants are presented with photos or video-clips of emotional expressions and are asked to imitate the facial expression. In others, participants may be asked to produce a facial expression appropriate to a particular scenario (e.g. when they are feeling sad or in response to a verbal label, e.g. MacDonald et al. 1989; Volker et al. 2009). There are also a number of studies that look at the production of spontaneous and naturalistic facial expressions of emotion. For example, Sebe et al. (2007) covertly filmed participants while they were presented with emotional stimuli, and Zeng et al. (2006) used naturalistic observation, recording facial expressions produced during conversations with an experimenter. A problem with this methodology is the difficulty of ensuring that all participants produce sufficient and similar content to elicit emotional facial expressions. This approach would therefore not work well for individuals with ASD who may have reduced conversational skills relative to a control group (Capps et al. 1998). An alternative method to elicit facial expressions in deaf signers is to use a short narrative and question–answer paradigm (Hendriks 2007). Hendriks argued that the benefit of using narratives when assessing facial expression production is that the topic, content and length of discourse can be controlled among individuals. Another advantage is that it reduces demands on memory. For these reasons, we adopted a similar methodology, using the BSL Narrative Production Test (BSLPT: Herman et al. 2004) to explore how deaf individuals with ASD and deaf TD controls compared in their production of facial expressions when using sign language. The BSLPT has been previously used in research contexts to explore language skills in deaf children with specific language impairment (Herman et al. 2014) and an adapted version has been used with deaf children who use spoken language (Jones et al. 2016).

The focus of this study was on the production of facial actions in describing an event using sign language, where paralinguistic (emotional and prosodic) expressions were likely to be elicited.^1^ There are two hypotheses: the first (the null hypothesis) is that it is possible that there will be no effect of ASD status on deaf children’s production of facial expressions in a BSL narrative task. This could occur if exposure to sign language and the need for deaf people to attend to facial actions may provide some protection against anomalies in emotional expression production that have been reported for hearing children with ASD. An alternative hypothesis is that emotional processing deficits associated with ASD may be more critical in determining emotional expression production which will not be improved by increased attention to faces for deaf children with ASD. In that case, given that hearing individuals with ASD have both reduced quantity and quality of emotional facial expressions relative to TD controls (Yirmiya et al. 1989; Volker et al. 2009; Brewer et al. 2016) and impaired prosody in production of speech (Peppé et al. 2006; Hubbard and Trauner 2007; Hubbard et al. 2017), we would expect the deaf ASD group to produce fewer and less appropriate facial expressions than deaf TD controls when producing a BSL narrative.

## Method

### Participants

Twelve TD deaf individuals were recruited from deaf schools across the UK. Ten deaf individuals with ASD were recruited from the National Deaf Child and Adolescent Mental Health Service, where they had received a diagnosis of ASD from a specialist multidisciplinary social and communication disorders clinic for deaf children, using an adapted version of the Diagnostic Interview for Social and Communication Disorders (DISCO: Wing et al. 2002); a nonverbal battery measuring cognition (Leiter-R: Roid and Miller 1997) and a play assessment. A detailed method of assessing and diagnosing this population was used, due to the challenges associated with diagnosing deaf children with ASD, namely the lack of a gold standard for assessment, the heterogeneity of deaf populations and the overlap between certain behaviours in both deafness and ASD; for instance, not responding to their name being called (Mood and Shield 2014). This battery of tasks was used as part of the standard protocol to assist with diagnosis in this complex population and rule out other conditions. The ASD group was made up of two individuals with a diagnosis of Asperger’s syndrome and the remaining eight had diagnoses of childhood autism. Diagnosis met the criteria in the Diagnostic and Statistical Manual of Mental Disorders, Fourth Edition (American Psychiatric Association 1994). At the time of recruitment, this was the most comprehensive assessment for deaf individuals with ASD in the UK.

All participants had bilateral severe or profound sensorineural hearing loss. Use of amplification was similar across both groups [ASD group: cochlear implant (5), hearing aids (4) and unaided (1); control group: cochlear implant (4), hearing aids (5) and unaided (3)]. In order to meet the inclusion criteria for the study, participants needed to be able to communicate using sign language at least at a phrasal level. One child in each group was a native signer with deaf parents, the remaining participants were all from hearing families. All participants were in bilingual education environments and were exposed to BSL in the classroom at the time of testing, which ensured greater consistency in signing abilities.

The groups were matched for chronological age, nonverbal intellectual ability using the Raven’s Standard Progressive Matrices (SPM: Raven et al. 1998) and BSL comprehension using the BSL Receptive Skills Test (BSLRST: Herman et al. 1999). Independent sample t tests indicated no significant difference between the groups in chronological age [t (21) = − .85, p > .40], nonverbal ability [t (21) = .15, p > .78] or BSL comprehension [t (20) = − .25, p > .94].

The Social Responsiveness Scale (SRS: Constantino and Gruber 2005) is a short checklist, which was given to teachers of all participants to further confirm the diagnosis of ASD. Higher scores on the SRS indicate greater severity of social impairment; T-scores below 60 are considered normal, T-scores between 60 and 75 indicate clinically significant impairment in the mild to moderate range, and T-scores above 75 indicate severe social impairment. As such, T-scores ≥ 60 are used to indicate that a diagnosis of ASD may be appropriate (Moul et al. 2014). In support of the independent diagnostic criteria for ASD in the experimental group, the SRS confirmed significant differences between the deaf ASD group and the deaf TD control group [t (21) = − 6.38, *p* < .001). Ethical approval was obtained from the ethics committee at University College London and the National Research Ethics Service, which is the ethical body for the NHS.

## Materials and Procedure

### Facial Expressions in the BSLPT

The BSL Production Test is a standardised test of sign language production for deaf children aged 4–11 (Herman et al. 2004). It requires the respondent to produce a coherent narrative in BSL by re-telling a 3-min, language-free video scenario with two child protagonists silently acting out a story. While the story is fairly complex, the test has been normed on deaf children as young as 4 years old who have been able to produce an appropriate narrative. The scenario was designed to elicit a range of lexical, morphological, grammatical and pragmatic features. The gist of the story is that the boy demands various food and drink items from the girl at different points in time, until she tricks him by putting a spider in a sandwich and giving it to him (see “Appendix” section). Retelling requires a signer to depict not only the actions of the protagonists but also their emotions which change through the scenario. The signers retold the story to the experimenter who is a hearing native BSL user. For the purpose of this study, adult native BSL signers determined where in the story a specific facial expression would be expected in a naturally signed re-telling of the story, to create a scoring template of emotional facial expressions. Sometimes, but not always, identification instances coincided with manual action representing a lexical emotion label. For example, the ‘demand’ facial expression would typically occur simultaneously with the manual sign for DEMAND. However, there were other instances where the emotional responses of the protagonists needed to be incorporated without lexicalisation. For example, the facial expression for ‘surprise’ would be expected in the re-telling of the scenario event where a protagonist found a spider in his mouth—even though this re-enactment need not include the manual sign SURPRISE (Table 1).

**Table 1:** Shows groups were matched on age, nonverbal intellectual ability and BSL comprehension

Group	Statistic	Age (year:month)	Raven SPM Raw score	BSLRST Raw score	SRS
TD (n = 12)	Mean	12:3	28.4	93.9	4.8
	SD	2:5	9.3	19.6	3.7
	Range	8:5–16:5	13–40	56–125	0–14
ASD (n = 10)	Mean	13:1	28.0	95.7	68.3
	SD	2:5	10.8	25.2	34.4
	Range	9:0–17:0	10–46	56–123	26–141

They differed on the SRS, a measure of ASD symptomology

Raters determined that the two actors produced the following six facial expressions during the video: demand, refusal, annoyance, surprise, mischief and disgust. Two of these are considered to be basic universal emotions: ‘disgust’ and ‘surprise’ (Ekman 2000). Measured variables were: number; timing; quality of facial expressions in the BSL narrative produced by the children, in relation to those expressions as identified by the raters. There were 16 points in the video scenario for which raters identified specific facial expressions which would be expected in re-telling the story.

Table 2 shows the number of emotional facial expressions that were present in the original acted scenario. A similar scoring system to McIntire and Reilly (1988) was used, where quantity and quality of facial expressions were coded. Within each expression the quantity of facial actions was scored. The scoring system was akin to the Facial Action Coding System (FACS), targeting the key facial actions in emotional expressions of BSL. The FACS is a system designed for human observers to describe changes in the facial expression in terms of visually observable activations of facial muscles (Ekman and Friesen 1978; Pantic and Rothkrantz 2004). FACS training is to enable the expert FACS user to distinguish differences between and within seven facial expressions. Here, the aim was different: it was to identify a (small) number of possible expression categories that fitted the narrative produced, and to use these to score respondents facial actions. However the FACS principles (identify the appropriate muscle groups used from video clips) underpinned the method for assessing the number and quality of facial actions—both in identifying the expressions used by the model storytellers (‘gold standard’, see below) and in assessing the quality of the expressions produced by respondent children. The experimenter met with a linguist specializing in sign language to discuss developing a suitable scoring system and then subsequently went on to train the second rater in how to use it.

**Table 2:** Number of emotional expressions produced by the characters in the BSLPT video

Affective expression	Number of times produced in narrative	Character
Demand	4	Boy
Refusal	4	Girl
Annoyance	3	Girl
Surprise	1	Girl
Mischief	2	Girl
Disgust	2	Boy

## Administering and Scoring BSL Retelling

Using BSL, the experimenter instructed participants to watch the video on a laptop computer and to re-tell the story afterwards, emphasising that they should include as much detail as possible in their re-tellings. The experimenter then left the room. After they produced their narrative, they were asked three questions to test their general understanding of the scenario. Narratives produced by the participants and responses to questions were video recorded for later analysis.

### Acquiring Gold Standard Measures of Expressions in the BSLPT Retelling: Adult Signers

Two deaf adult native signers watched the BSLPT event and produced a re-telling on video. Adult native signers were selected because they have acquired sign language from birth and therefore represent best case examples for this production task (McIntire and Reilly 1988; Costello et al. 2006; Cormier et al. 2013). The facial actions within the signed narratives of the two adults were analysed and a maximum score was created for each expression. For example, ‘demand’ consists of five facial action targets to be produced correctly (see Table 3). The analysis of these adult facial actions within a given expression formed the baseline against which the children’s videoed data were scored. The adult re-tellings were used as a gold standard to compare the childrens’ facial expression scores. All video responses were annotated using sign language annotation software: EUDICO Linguistic Annotator (ELAN; Lausberg and Sloetjes 2009).

**Table 3:** Emotional expressions in the BSLPT and their corresponding facial actions

Affective expression	No of facial action targets based on the FACS coding of the adult native signers
Demand	(1) Head push forward (2) Furrow or raise eyebrows intensely (3) Widen eyes (4) Downturned closed mouth 5) Blink slowly
Refusal	(1) Head shake (2) Furrowed eyebrows (3) Frown (4) Wrinkled nose
Annoyance	(1) Roll eyes (2) Raise eyebrows (3) Shrug shoulders
Surprise	1) Widen eyes (2) Raise eyebrows
Mischief	(1) Look from side to side (2) Raise shoulders (3) Raise eyebrows
Disgust	(1) Raise eyebrows (2) Widen eyes (3) Mouth wide open (4) Furrowed eyebrows (5) Tilted back, body or head

## Scoring the Children’s BSL Narratives

**Fig. 1:**
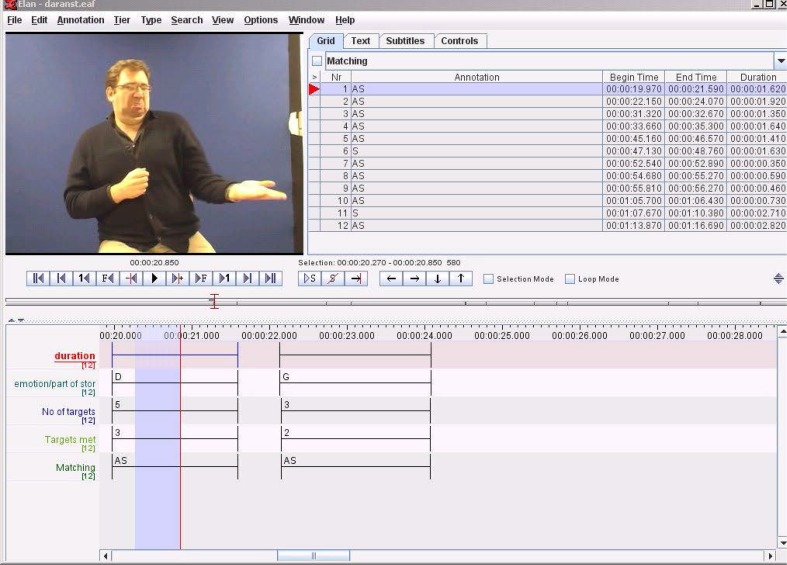
Example of one of the deaf adult signers producing the ‘demand’ facial expression using ELAN

Two raters scored the childrens’ video narratives. The first rater (the experimenter) was a native sign language user and was aware of the participant group status of the individuals. These ratings were then sampled by a deaf native sign language user who was blind to group membership. The second rater scored 20% of the BSLPT videos (10% from each group). Both raters were required to identify when each expression was produced and label it in the ELAN video file (see Fig. 1 for an example of the ELAN transcript from the adult video). Then they scored the number of appropriate facial actions within each expression in accordance with the scoring criteria in Table 3. Finally, they rated each expression for similarity to the adult model expressions, specifically whether the facial actions were ‘identical’, ‘similar’, ‘missing’ or ‘miscellaneous’. The adult re-tellings were seen alongside the children’s actions in ELAN on the computer screens for direct comparison. If the participant produced some but not all of the facial action targets for a given expression then this was scored as ‘similar’. So, if 2/3 facial action targets were produced for ‘annoyance’ but the signer did not shrug the shoulders (see Table 3 for further detail) this was counted as ‘similar’. If no targeted facial action was produced at a expected point in the narrative, then this was coded as ‘missing’. Expressive facial actions, which did not correspond with those produced by the model, were coded as miscellaneous.^2^ Raters scored the videos independently for facial expression similarity, and then reached a consensus through detailed discussion to agree the final rating used in this analysis. Intraclass correlations between the independent ratings showed overall agreement between raters was .94.

Each participant’s score was calculated on the basis of number of productions and how many facial actions they produced for each expression relative to the maximum possible score for that expression. For example, one participant produced three ‘demand’ facial expressions in their narrative. The maximum (gold-standard) score is five facial action targets for each ‘demand’ expression and this participant scored 11 out of 15 for ‘demand’ overall, missing four facial actions in total. Overall scores for each participant were then converted into percentages, by calculating the total number of facial actions divided by the maximum number of facial actions that could have been produced. So the participant described above produced 32/44 facial actions, and a facial action score of 72%. In this way, the child participants were compared on their number of overall facial actions produced (for all expressions combined) relative to the adult gold standard measures.

## Results

The BSLPT includes three screening questions to ensure participants had attended to and understood what happened in the scenario. The questions are asked in BSL. The translated English equivalents are: (1) what was on the tray? (2) Why did the boy throw the spider? and (3) why did the girl tease the boy? Each question is worth 2 points, and there is a maximum obtainable score of 6 points.

There were no differences between groups for story comprehension as measured by the BSLPT content questions [t(22) = − .69, p > .05, $\eta _{p}^{2}$ = − 0.29), indicating that both groups had equivalent understanding of the narrative.

Non-parametric Mann Whitney tests were used to compare the groups. The groups did not differ significantly on the length of narratives produced, although the ASD group had shorter narratives [U(25) = 41, p > .05, $\eta _{p}^{2}$ = − 0.58, (median duration in minutes.seconds: ASD: 2.52, TD: 3.23)].^3^ There was no significant difference between the TD and ASD groups in the number of overall facial action targets produced [U(22) = 53, p > .05, $\eta _{p}^{2}$ = − 0.92]. Because of the natural variation of expression in facial actions in BSL (Sutton-Spence and Woll 1999), the deaf adult signers did not produce the maximum number of facial actions each time for each expression. Therefore the overall number of facial action targets produced by the deaf adults (using Table 3 as a scoring guide) was 68.2% (median value). For the TD group this value was 55.8% and for the ASD group 45.4%.

## Group Differences

**Fig. 2:**
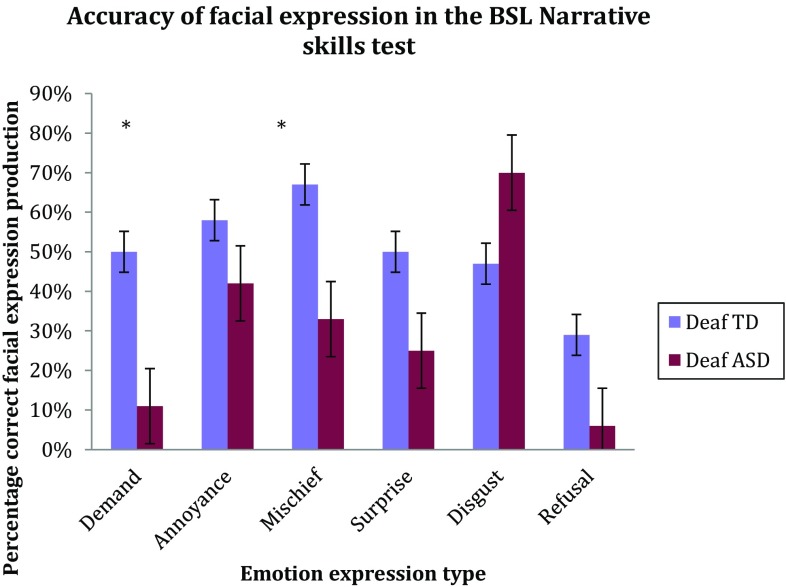
Production of specific, appropriate emotional facial expressions for ASD and TD groups

**Fig. 3:**
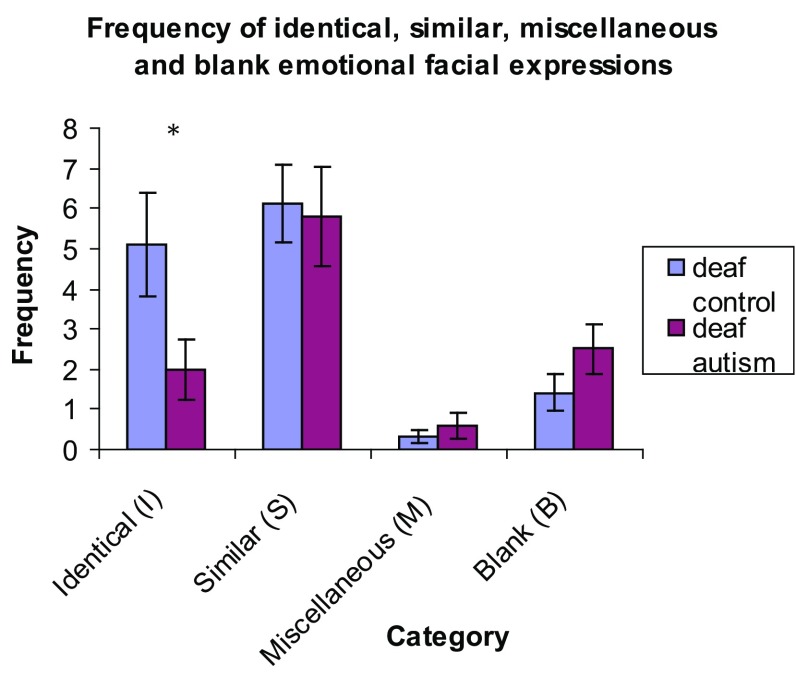
Quality of emotional facial expressions produced in the BSLPT

Figure 2 shows how both groups fared with specific emotional expressions. The TD group produced significantly more facial actions than the ASD group for ‘demand’ [U(22) = 29.5, p < .05, $\eta _{p}^{2}$ = − 0.96] and ‘mischief’ [U(22) = 35.5, p < .05, $\eta _{p}^{2}$ = − 0.61] (see Table 4 for means and standard deviations). There were no significant group differences across the other four expressions. There were no significant differences between groups for the number of missing facial expressions [U(22) = 98.5, p > .05, $\eta _{p}^{2}$ = 0.77). However, the frequency of face expressions rated ‘identical’ to the gold standard differed significantly between groups, with the TD group producing a greater number of identical expressions compared to the ASD group [U(22) = 37.5, p < .05, $\eta _{p}^{2}$ = − 0.70] (Fig. 3). There were no significant differences between groups in the number of similar or miscellaneous facial expressions produced.

**Table 4:** Mean and standard deviation of facial action targets for each emotion

	Deaf ASD: M (SD)	Deaf control: M (SD)
Demand	23.6 (21.2)	48.1 (28.4)
Annoyed	41.5 (25.1)	47.6 (33.6)
Mischief	42.6 (33.0)	63.8 (36.1)
Surprise	47.5 (44.7)	37.5 (37.6)
Disgust	67.3 (27.8)	49.4 (21.5)
Refusal	29.3 (30.0)	35.1 (34.0)

The relationship between SRS severity and facial expression production was explored. There were significant correlations between SRS scores and two expressions: ‘annoyance’ r (11) = − .82, p < .05, and ‘mischief’ r(11) = − .77, p < .05. That is, higher SRS scores (more reported autistic traits) were associated with fewer ‘annoyance’ and ‘mischief’ facial actions.

To test whether differences in general sign language or narrative abilities might account for differences in emotional facial expression production, scores on the BSLPT were compared (see Herman et al. 2004, for example of BSLPT scoring system). Scores were calculated to determine how participants in both groups fared with narrative content (this was scored by awarding a point for explicit mention of each of 16 narrative episodes, maximum score = 16), narrative structure (scored for orientation, complicating actions, climax, resolution and sequence, maximum score = 12) and grammar (scored by the correct use of five classes of morphological inflections, maximum score = 30, reflecting the number of different verb forms targeted and for role shift). Participants’ scores on these three parameters were marked using the BSLPT scoresheet.

A Kruskal–Wallis non-parametric test was calculated for raw scores on each test parameter. No differences were found between groups for narrative content (H(1) = .132, p = .71), narrative structure (H(1) = .89, p = .018) or grammar (H(1) = 2.132, p = 1.44).

## Discussion

This is the first study to investigate the frequency and quality of facial expressions produced during a sign language narrative in deaf children with and without ASD. No differences were found between groups in terms of the total number of facial expressions produced, but there was a significant difference in the quality of facial actions produced. Deaf children with ASD compared with TD deaf children produced fewer expressions which corresponded to those produced by adult deaf signers when they retold the story. When individual expressions were analysed, the groups did not differ significantly on four of the six facial expressions scored, however deaf children with ASD produced significantly fewer facial actions for ‘demand’ and ‘mischief’ relative to deaf controls.

**Fig. 4:**
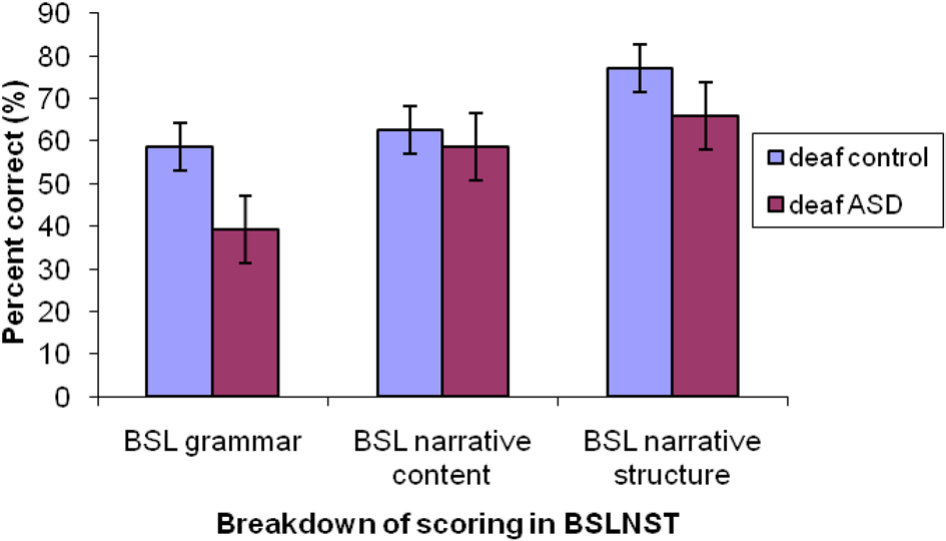
Narrative content, structure and grammar scores for the TD and ASD groups

The reduced quality of facial expressions in the deaf ASD group was broadly consistent with the finding of emotion production impairments (MacDonald et al. 1989; Volker et al. 2009; Grossman et al. 2013) in hearing groups with ASD (Fig. 4).

Our findings do not support the hypothesis that emotional facial expressions might be preserved in deaf people with ASD because of a protective effect afforded by deafness and sign language use. Instead, the results suggest that deaf signing children with ASD show impairments in emotional facial expression production because deficits in emotion processing and theory of mind are central to autism and disrupt this ability in signers, as they do for users of a spoken language. Further research is needed to support this initial hypothesis. The current study shows impairment in the quality of some facial expressions in deaf ASD children, analogous to ‘odd’ expressions produced by hearing individuals with ASD (MacDonald et al. 1989; Volker et al. 2009). It is not possible to identify from our results the underlying cause of these production impairments. A number of studies with hearing ASD participants have noted the high co-occurrence with autism of alexithymia [a personality trait characterized by a marked dysfunction in one’s own emotional awareness (Cook et al. 2013; Trevisan et al. 2016)]. Brewer et al. (2016), in a study with hearing ASD participants and controls, ruled out both a reduced proprioceptive awareness of facial muscles and a reduced motivation to produce information on the face as causes, arguing that atypical cognitive representations of facial emotions are more likely to underlie poor production of emotional expressions.

The severity of social communication impairment scored using the Autism Diagnostic Observation Schedule, (ADOS: Lord et al. 1999) has been found to correlate with vocal and facial ‘awkwardness’ (Grossman et al. 2013). We also found correlations between severity of social communication impairment in deaf children (as measured by SRS) and scores for facial expressions. In our study this correlation was for the expressions ‘annoyed’ and ‘mischief’. SRS scores were not associated with the ‘demand’ expression. This may be a result of its decreased salience relative to the other expressions. It has been proposed that impairments in emotion recognition in ASD are greater for more subtle expressions of emotion (Griffiths et al. 2017).

Deaf children with ASD did not differ from controls in their ability to produce a coherent and linguistically correct narrative. The duration, content, structure and use of grammatical features in their narratives were remarkably similar to typically developing deaf children. Therefore group differences in production of targeted emotions were unlikely to reflect differences in linguistic skills or in understanding the model scenario. Both groups were matched on their BSL receptive skills and they did not significantly differ in performance on the narrative content, structure or grammar scales of the narrative production test. Nevertheless the deaf ASD group had poorer performance on the grammar scale, which may suggest that this subgroup have superior comprehension skills in comparison to production of BSL. Further research could explore this hypothesis. In both groups, nine participants used hearing aids and cochlear implants, so there is a possibility that they were receiving auditory input as well as visual and signed information. This may suggest that it is sign language exposure rather than deafness per se that has led to increased attention to the face and better communication skills in this population, however this question remains currently unexplored.

It is of interest that the deaf ASD group produced more facial actions for the canonical expression ‘disgust’. Volker et al. (2009) also found a non-significant trend where hearing individuals with ASD produced more examples of this expression than TD controls. Our finding could have been influenced by the fact that ‘disgust’ occurs at the end of the narrative during the climax of the story. Endpoints of narratives are usually recalled more accurately due to the recency effect and the because endpoints often mark the semantic climax of a story (Poulsen et al. 1979; McCabe and Peterson 1990). It has also been argued that ‘disgust’ is a particularly salient emotion having evolutionary significance as a response to potential contamination (Rozin and Fallon 1987). It was one of the most distinctive expressions produced in the scenario, which may have meant it was picked up more readily by the ASD group, whose expression processing skills may be more marked for less intense emotions (Wong et al. 2012; Griffiths et al. 2017). It would be of interest to further explore whether the intensity of emotions produced in the scenario had any impact on their production during the re-telling.

When the adult deaf signers modeled the narrative, they produced not only classic emotional expressions (‘disgust’, ‘surprise’, ‘annoyance’ (anger)) but also expressions which could involve both emotion and other cognitive and communicative features (‘mischief’, ‘demand’ and ‘refusal’). This is likely to be because the narrative itself involved a series of communicative events including a scenario in which one character deceives another (a girl hiding a pretend spider inside a sandwich that she knows a boy will eat). Indeed, it could be argued that a theory of mind (ToM) is required to fully understand the narrative. Hearing individuals with ASD have been shown to have deficits with ToM, and often fail false belief tasks (Baron-Cohen et al. 1993; Dennis et al. 2000). Although deaf children from hearing families who do not have exposure to language at an early age can also be delayed in ToM compared to TD hearing children, the delay is less severe than it is for hearing children with ASD (Peterson and Siegal 1999; Woolfe et al. 2002; Schick et al. 2007). It is possible then, that some participants failed to use appropriate facial acts in contexts where they did not fully understand the ToM elements of the story. If ToM is more impaired in the deaf ASD than the deaf control participants this may explain some differences in the use of facial expressions between the groups. For example, the deaf ASD group produced significantly fewer “mischief” facial acts; in the context of the narrative, understanding “mischief” involves understanding the characters’ mental states, as it refers to the girl’s deception in hiding the spider in the sandwich to trick the boy, who knows nothing of it. It is also possible that group differences in using the facial expression “demand” could be explained by differences in ToM understanding, at least in so much as understanding others’ desires has been described as a mentalising skill (Wellman et al. 2000). It is notable that ASD children produced more examples of ‘surprise’, ‘disgust’, ‘annoy/anger’ which are universal emotional facial expressions described by Ekman and Oster (1979) and relate to more instinctive emotional reactions rather than the mental states normally associated with ToM. However, they differed in the quality of facial acts across all categories. It is difficult to explain this reduction in quality in terms of understanding the ToM elements of the narrative. Instead, the quality of facial expressions produced in deaf ASD participants seems more likely to be linked to general deficits in production of emotional expressions in ASD (Blair 2003). The third question (asking why the girl teased the boy) hints at whether or not participants may have had ToM. Some were able to state ‘the girl was angry’ vs. ‘the girl did not like spiders’. However, this would need further clarification for a follow up study. We did not directly test narrative understanding in terms of ToM in this study, but our data suggests that this would be an interesting approach to take in future studies.

A strength of this study is the use of an ecologically-valid, narrative methodology to elicit facial expressions; this contrasts with more artificial techniques sometimes used where participants are asked to copy facial expressions. This did however mean that only a narrow range of expressions were explored, and did not include all of Ekman’s classic examples of facial expressions (Ekman and Oster 1979). We would recommended further studies using other narrative tasks designed to elicit more of the Ekman-categorised ‘universal’ facial emotions so groups can be compared across a wider range of emotions, which could also differ in the strength of the emotion portrayed. We also had a relatively small sample size due to the difficulty in recruiting deaf ASD participants. Any null group differences may be attributable to the lack of statistical power and caution should therefore be taken in extrapolating our findings to the wider deaf ASD and deaf TD populations. As recruitment was largely opportunistic, there was a discrepancy between the age the BSLPT is designed for (ages 4–11) and the age of the participants in the sample (ages 8.5–17). Future research on younger deaf children with ASD on this measure would be useful to demonstrate whether performance is poorer at a younger age as a result of less sign language exposure or due to developmental delay. In addition, the use of relatively large age ranges, e.g. from early childhood to adolescence, make it difficult to compare results between different studies to determine whether people with ASD show any developmental differences in their narrative production skills.

One relevant caveat of the current study is the lack of assessment of imitation or motor skills in deaf children with ASD. It is well known that children with ASD often have a range of motor deficits (Ghaziuddin and Butler 1998; Jansiewicz et al. 2006; McPhillips et al. 2014). This deficit in motor skills may have an impact on the production of BSL signs in deaf children with ASD and subsequently hinder the emergence of social communication skills.

Facial expressions in BSL convey both emotion and linguistic meaning. The present study focused on emotional facial expressions. The production of BSL linguistic facial expressions will be reported in a future paper. We expect that there will be differences in how both groups use emotional and linguistic facial expressions since linguistic expressions are more rule-governed, and may be easier for individuals with ASD to learn.

The findings from the current study on facial emotion production lend some support to the hypothesis that deaf children with ASD have difficulties similar to those of their hearing counterparts. However, there is still much more research to be done in this field. In particular, research carried out with hearing individuals with ASD exploring their ability to be taught and use facial actions in BSL could inform whether the use of sign language helps facilitate attention to the face and improve communication skills.

Impairments in facial expression production in deaf individuals with ASD are likely to impact on interactions with other deaf signers which may further exacerbate social communication (Shriberg et al. 2001; McCann and Peppé 2003; Paul et al. 2005). Further research is needed, focusing on interventions for this group, using adaptations to evidence-based approaches for hearing children with ASD (Watkins et al. 2015). In the first instance, deaf children with ASD should be taught to discriminate between emotional facial expressions in BSL and to produce them appropriately. These basic emotion recognition skills could then be developed further to enhance social communication. For example, deaf children with ASD could benefit from attending social skills groups, which are facilitated in sign language to foster social and communication skills. In addition, deaf children with ASD should be assessed during naturalistic social situations when communicating in BSL to compare whether self generated production of emotional facial expressions differ to those elicited from observations or narratives. This would lead to a greater awareness of the use of emotional facial expressions in BSL in this population and subsequently inform the development of further appropriate ecologically valid interventions. Interventions for hearing children with ASD that are focused on emotional prosody emphasise using stronger intonation cues e.g. lower pitch, longer tempo and louder amplitude (Wang et al. 2006; Matsuda and Yamamoto 2013). In a similar manner, teaching of non-manual prosodic markers in BSL could be made more explicit to deaf children with ASD so that facial actions are exaggerated, made more salient or taught more intensively to facilitate the learning and use of facial expressions. Alternatively deaf children with ASD could be taught compensatory cognitive or linguistic strategies (Rutherford and McIntosh 2007), for example, always producing the manual sign for the emotion that they are referring to in addition to the non-manual facial action to clarify meaning.

This study shows that deaf children with ASD show subtle differences in terms of their production of emotional facial actions during narrative retelling. These data are an important first step in documenting differences in sign language production for deaf children with ASD, with affected aspects of communication relating to emotion and mentalising about the internal state of others.

## Appendix: Story Episodes

1. The girl brings in a tray of food and drink
2. The boy is watching TV.
3. The girl helps herself to sweets, which the boy demands (using an outstretched arm movement and an insistent facial expression) and she gives them to him.
4. Episode (3) is repeated with a cake.
5. Episode (3) is repeated with a drink.
6. The girl sees a spider.
7. She tiptoes over to pick up the spider (whilst the boy continues to watch TV).
8. She makes a sandwich by placing the spider between two pieces of bread.
9. She pretends to eat the sandwich.
10. The boy demands the sandwich.
11. The girl hands over the sandwich to the boy.
12. The boy bites the sandwich (and realizes there’s a spider inside).
13. He takes the spider out of his mouth.
14. He chases the girl round the room.
15. He throws the spider at the girl.
16. Additional information provided, e.g. the boy is lazy or the spider is horrible

## Footnotes

1 The production and comprehension of face acts intrinsic to linguistic processes is the topic of a separate report.

2 We excluded from this analysis facial expressions that were clearly linguistic aspects of the BSL production, such as grammatical markers.

3 This scoring procedure only takes account of targeted facial actions produced at precise narrative points by the children: other facial actions occurring during the retelling were not scored.
